# APP‐mediated intracellular signaling rescues sleep impairment and blood–brain barrier leakage in Alzheimer's disease mouse model

**DOI:** 10.1002/alz.71134

**Published:** 2026-02-03

**Authors:** Clémentine Puech, Anjana Sadanand, Neil Coleman, Marilyn Ikhane, Mohammad Badran, Rong Wang, David Gozal, Angèle T. Parent

**Affiliations:** ^1^ Department of Pediatrics School of Medicine University of Missouri Columbia Missouri USA; ^2^ Present address: Clémentine Puech, Group of Precision Medicine in Chronic Diseases. Hospital Nacional de Parapléjicos. IDISCAM Toledo Spain; ^3^ Department of Molecular Medicine University of South Florida Tampa Florida USA; ^4^ Department of Medical Pharmacology and Physiology School of Medicine University of Missouri Columbia Missouri USA; ^5^ Departments of Pediatrics and Biomedical Sciences and Office of the Dean Joan C. Edwards School of Medicine Marshall University Huntington West Virginia USA

**Keywords:** AD mouse models, adenylate cyclase, amyloid precursor protein signaling, astrocytes, blood‐brain barrier, cognition, sleep

## Abstract

**INTRODUCTION:**

Amyloid beta peptide (Aβ) accumulation in the brain is an Alzheimer´s disease (AD) hallmark. Sleep disturbances hamper Aβ production and clearance, thereby exacerbating the Aβ burden. The mechanisms involved remain unclear. We reported that amyloid precursor protein (APP), the Aβ source, possesses intracellular signaling that attenuates Aβ production and prevents cognitive decline in AD mice. Our follow‐up study assessed whether enhancing APP‐mediated signaling affected sleep.

**METHODS:**

We expressed a membrane‐tethered APP intracellular domain (mAICD) and a variant lacking the Gα_S_‐interacting site in AD mouse brains. Sleep patterns, cognitive behaviors, blood–brain barrier (BBB) integrity, and gliosis were examined.

**RESULTS:**

Sleep, BBB integrity, and memory were strongly correlated. mAICD expression rescued sleep and cognitive function impairments, prevented BBB leakage, and promoted astrocyte redistribution surrounding the neurovascular units in AD mice. Gα_S_ interaction with mAICD was critical.

**DISCUSSION:**

APP‐mediated signaling plays a key role in regulating sleep, maintaining BBB integrity, and preserving memory in AD.

## Highlights

- Sleep, cognition, and BBB permeability are tightly interconnected.
- mAICD brain expression restores sleep and prevents vascular impairments in AD mice.
- mAICD interaction with Gα_S_ is necessary to preserve BBB, sleep, and cognition.
- mAICD redistributes astrocytes in NVUs through Gα_S_ signaling.

## 1 BACKGROUND

Alzheimer's disease (AD) is a common neurodegenerative disorder characterized by progressive cognitive decline, including memory loss, language deterioration, visuospatial deficits, emotional problems, decreased motivation, and impaired daily functioning.^1^, ^2^, ^3^ Sleep disturbances are also frequently observed in AD patients, even in the early stages of the disease.^4^, ^5^, ^6^ Indeed, the emergence of fragmented sleep, as illustrated by multiple and recurring arousals, can be an early indicator of memory decline and is currently viewed as an independent risk factor of AD.^4^, ^7^, ^8^ There is a bidirectional relationship between sleep disturbance and AD pathology, but their independent contributions are yet to be elucidated.^4^, ^9^, ^10^, ^11^ Notwithstanding extensive research efforts, available therapies remain limited and primarily focus on symptom relief.^12^ There is a pressing need to improve our understanding of the pathogenesis of the disease to enable the development of disease‐modifying treatments.

AD is characterized by two fundamental hallmarks, extracellular amyloid beta peptide (Aβ) accumulation and aggregated neurofibrillary tangles containing hyperphosphorylated tau protein (reviewed elsewhere^12^, ^13^, ^14^). These pathological changes are linked to neuronal atrophy and synaptic loss along with chronic brain inflammation, which can promote blood–brain barrier (BBB) dysfunction (reviewed elsewhere^2^, ^15^, ^16^). It has been suggested that BBB breakdown may precede the emergence of cognitive decline and neurodegeneration.^16^ The BBB is a highly regulated neurovascular unit (NVU) that plays a crucial role in the performance and protection of the brain by allowing a selective passage of nutrients while preventing the entry of potentially neurotoxic substances. Several studies demonstrated that BBB dysfunction reduced Aβ clearance.^17^, ^18^ It has been proposed that this condition creates a damaging feedback loop where reduced clearance causes Aβ accumulation, which further compromises BBB function, ultimately giving rise to cognitive impairments and sleep disturbances.^4^, ^7^, ^19^

**RESEARCH IN CONTEXT**

1. **Systematic review**: A bidirectional link between sleep disruption and AD pathology has been suggested. Poor sleep reduces Aβ clearance and exacerbates Aβ burden, while elevated Aβ further impairs sleep. Although Aβ accumulation, sleep perturbations, and BBB dysfunction are increasingly recognized, other APP metabolites’ contributions remain undefined. We previously reported that brain expression of mAICD prevented cognitive decline in AD mice. We followed up on these findings.
2. **Interpretation**: We observed that mAICD expression restores sleep continuity, reduces BBB permeability, promotes astrocyte redistribution, and improves memory in AD mice. Gα_S_ interaction with mAICD is required, revealing a novel role of APP‐mediated signaling with sleep and neurovascular regulation in AD.
3. **Future directions**: To further identify potential translational intervention, we will need to assess whether mAICD efficacy is significant at later disease stages. The cell‐type‐specific pertinence of mAICD expression needs clarification, as the sexual dimorphism in disease outcomes is noteworthy.

Aβ is produced through the successive cleavage of the amyloid precursor protein (APP) by β‐secretase and then γ‐secretase, which are known to play a key role in AD pathology.^20^, ^21^, ^22^ APP can also be cleaved by α‐secretase within the Aβ amino acid sequence, thereby preventing Aβ formation, leading to the non‐amyloidogenic pathway. α‐ and β‐secretases generate membrane‐bound C‐terminal fragments (APP‐CTFs) comprising the transmembrane and cytoplasmic domains. APP‐CTF cleavage by β‐ and γ‐secretases releases Aβ and the APP intracellular domain (AICD) from the membrane, which possesses a short half‐life because of its rapid degradation. We previously reported that retention of APP‐CTF at the membrane favored intracellular signaling that prevented Aβ generation.^23^, ^24^ Consistent with this observation, we found that expression of a membrane‐tethered APP intracellular domain construct (mAICD) in AD mouse brains reduced Aβ pathology and improved cognitive function. We further determined that mAICD expression engaged the heterotrimeric Gα_S_‐protein to activate a signaling cascade underlying these cognitive improvements.^24^ As a follow‐up study, we investigated whether mAICD and its Gα_S_‐associated signaling could modify the sleep pattern and BBB integrity in the amyloidogenic 5XFAD mouse model. We found that this well‐established murine AD model exhibited a profound alteration of the BBB integrity concurrent with reduced sleep continuity. Perinatal induction of mAICD expression in the brain rescued these impairments, an effect that was absent in a construct lacking the Gα_S_ interaction site. These findings highlight a novel role for APP‐CTF and its associated cyclic adenosine monophosphate (cAMP)/protein kinase A (PKA)‐dependent signaling in AD pathology.

## 2 METHODS

All experiments were approved by the Institutional Animal Care and Use Committee of the University of Missouri (9658‐33864) and the University of South Florida (10682). The study followed the Animal Research: Reporting of In Vivo Experiments (ARRIVE) guidelines. All efforts were made to minimize animal suffering and to reduce the number of animals required for the study.

Transgenic 5XFAD mice expressing five familial AD mutations (3XAPP [Swedish K670N/M671L, Florida I716V, and London V717I], and 2XPS1 [M146L and L286V] mutations) were used: Male and female 5XFAD mice and their wild‐type littermates B6SJL from the University of South Florida colony were utilized between the ages of 6 and 8 months. Polymerase chain reaction (PCR)‐amplified regions using specific primers for each strain were performed to confirm the genetic identity of the mice, as previously described.^23^ Animals were housed in a controlled environment with 12‐h light‐dark cycles (from 6 a.m. to 6 p.m.) at constant temperature (24 ± 0.2°C) with ad libitum access to food and water. All animals were allowed to recover and fully acclimate within the animal care facility for at least 7 days after they arrived from the University of South Florida at the University of Missouri.

### 2.1 Adeno‐associated virus (AAV) constructs and neonatal injection

**FIGURE 1:**
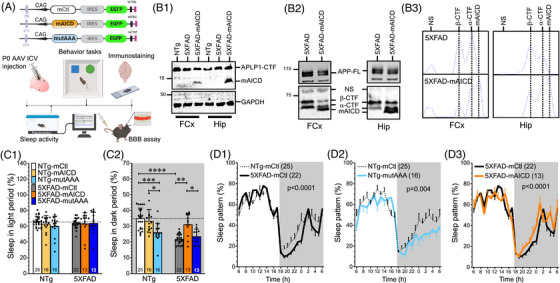
Amyloid precursor protein (APP)‐mediated signaling affects sleep patterns. (A) Experimental design of the study is represented as a schematic displaying adeno‐associated virus (AAV) constructs utilized in our study (membrane‐tethered control [mCtl], membrane‐tethered APP intracellular domain [mAICD], mutated mAICD [mAICD‐mutAAA] variant lacking the Gα_S_ interaction site), with which the neonates were injected in the intracerebroventricles. Six to 9 months later, sleep activity was recorded using cages equipped with a piezo sensor device. Next, the cognitive behavior tasks were performed using the elevated‐plus maze (EPM), and Novel Object Recognition (NOR). Blood–brain barrier (BBB) integrity was assessed. The mouse brains were collected for immunostaining in independent cohorts. (B1) Western blot analysis from frontal cortex (FCx) and hippocampus (Hip) lysates is shown for the NTg, 5XFAD, and 5XFAD‐mAICD, using the APLP1‐CT11 rabbit Ab to identify the CT11‐tagged mAICD. (B2) Accumulation of APP‐CTF metabolites is observed using APP‐CTM1 rabbit Ab. (B3) Relative protein densities from the CTM1 blot are shown as plot lines in brain lysates from 5XFAD mice expressing mAICD compared to 5XFAD control. (C) Percentage of sleep activity was analyzed during the total 12 h of (C1) light and (C2) dark phases. Data are shown as mean ± standard deviation (SD). Statistical analysis was performed using the ANOVA Kruskal‐Wallis test, followed by Dunn's multiple comparisons. * *p *< 0.05, *** *p *< 0.001, **** *p *< 0.0001. (D) Sleep pattern was analyzed every hour, averaged over a 2‐day period, and compared between (D1) NTg‐mCtl and 5XFAD‐mCtl, (D2) NTg‐mCtl and NTg‐mutAAA, and (D3) 5XFAD‐mCtl and 5XFAD‐mAICD. NTg‐mutAAA and 5XFAD mice exhibit a reduction of sleep during the dark period, which was rescued in 5XFAD expressing mAICD. Statistical analysis was performed on the 24 h period using two‐way ANOVA. Data are shown as mean ± SEM.

We proceed with the intracerebroventricular injection of various recombinant AAV constructs in pups for 8 to 16 h after birth. Our constructs were previously described^23^ (see also schematic in Figure 1A). We generated cDNA by PCR, which was inserted into a pTR‐UF22 vector, and a chimeric AAV serotype 2/8 hybrid recombinant gene delivery system consisting of *AAV2* inverted terminal repeats and *AAV8* capsid genes (AAV2/8). We used the following AAV constructs: pTR‐UF22‐CAG‐mCtl‐IRES‐EGFP construct of membrane‐targeted lipid‐raft control (mCtl), pTR‐UF22‐CAG‐mAICD‐IRES‐EGFP construct of membrane‐tethered APP intracellular domain (mAICD), and pTR‐UF22‐CAG‐mAICDmutAAA‐IRES‐EGFP construct of mAICD lacking the Gα_S_ interaction site (mAICDmutAAA or mutAAA (see details in Deyts et al.^24^). We also used a few naïve mice as controls. Briefly, cryoanesthetized neonates were injected with 2 µL of virus (2 × 10^10^ genome copies) into each lateral ventricle, followed by slow retraction of the needle. After injection, the pups were allowed to recover on a warming pad before returning to the home cage with the mother.

### 2.2 Sleep recording

Sleep/wake activity was monitored using a computerized piezoelectric system (PiezoSleep; Signal Solutions, Lexington, KY, USA), as described previously.^25^, ^26^ Briefly, a piezoelectric sensor was positioned beneath the mouse cage to detect changes in pressure. The system automatically assessed the intensity and duration of these pressure changes, classifying them as either wake or sleep states using a computerized scoring program (SleepStat; Signal Solutions, Lexington, KY, USA). The piezoelectric system was previously validated and demonstrated >90% accuracy compared to electroencephalogram/electromyography‐based sleep recording and scoring systems.^26^ Signals were extracted from short‐time pressure signal segments and classified automatically every 2 s. Sleep scoring was conducted continuously for 48 h under normal conditions.

### 2.3 Behavioral tests

To ensure unbiased results, behavioral assessments were carried out by two observers who were blinded to the treatment groups. Tests were performed in random order. The behavioral assessments encompassed the Novel Object Recognition (NOR) and Elevated Plus Maze (EPM) tests. Maze equipment for EPM and NOR was obtained from Maze Engineers (Cambridge, MA, USA). The recordings for EPM and NOR were captured from an overhead vantagepoint using a digitized video camera. All experiments were integrated with video software (Noldus Ethovision XT17 Software, Leesburg, VA, USA). The experimental setup was thoroughly cleaned and then wiped with 70% ethanol to prevent odor cues between each trial.

#### 2.3.1 Novel object recognition

The NOR test has been extensively described.^27^, ^28^ Briefly, this experimental paradigm is used to evaluate explicit memory based on the innate tendency of mice to explore novelty settings. For each trial, mice were placed in the center of a blue opaque open‐field plastic chamber. The NOR test is composed of three distinct phases. The habituation phase allows the mice to explore the open field twice for 10 min. During the second phase of 5 min, two identical objects are placed in the arena. During the last phase, one of the objects is replaced by a new object. The mice were allowed to freely explore the objects for 5 min. Positive exploration by the mouse was defined as touching the object with its nose. The tracking system analyzed and quantified the time spent exploring the objects. The total exploration time for both objects was recorded. Results were reported as preference scores using the following formula:

$$\mathrm{Preference}\;\mathrm{score}=\frac{\;\mathrm{Time}\;\mathrm{spent}\;\mathrm{near}\;\mathrm{novel}\;\mathrm{object}}{\mathrm{Time}\;\mathrm{spent}\;\mathrm{near}\;\mathrm{all}\;\mathrm{objects}}\;\times 100$$

Mice that did not explore objects were removed from the experiment. Animals were considered to have an explicit preference for novelty if their preference score was >50%.

#### 2.3.2 Elevated‐plus maze

The EPM is specifically designed to evaluate anxiety‐like behaviors in animal models. This task assesses two innate opposing behaviors in rodents: (a) the avoidance of open space exposures and (b) the inclination to explore new environments, as previously described.^28^ Mice with higher levels of anxiety tend to spend less time in the open arms of the EPM, displaying hesitation. Conversely, less anxious and impulsive mice tend to spend more time in open arms. The maze structure consists of an elevated cross positioned 56 cm above the floor, with two open arms and two closed arms extending from a central platform, forming a plus‐sign shape. During testing, the animals are placed in the central area, facing one of the open arms, and given 5 min to freely explore the maze. The time spent in open arms is measured by the software. We considered the central zone as part of the open arms.

### 2.4 BBB permeability

The BBB permeability assay was previously described.^27^ Briefly, animals were deeply anesthetized, and 3 to 5 kDa dextran–FITC (10 mg/mL; Sigma‐Aldrich, Catalog No.: 68059) was injected in the tail vein. After 20 min, mice were euthanized with CO_2_ and perfused with phosphate‐buffered saline (PBS), followed by brain harvesting, weighing, and homogenization in Tris‐HCl. After centrifugation, equal volumes of methanol were added to the supernatants, followed by another centrifugation. Fluorescence in the supernatants (triplicate samples per mouse brain) was then detected at 528 nm with an excitation wavelength of 485 nm using a microplate reader.^29^ Dextran concentration was calculated from a calibration curve, and tissue fluorescence values were normalized against fluorescence readouts from mouse brains without dextran.

### 2.5 Brain collection and immunostaining

Mice were anesthetized using isoflurane and subsequently transcardially perfused with PBS. The brains were then extracted and divided along the midline. One hemisphere was immediately flash‐frozen on dry ice and stored at −80°C. The other hemisphere underwent post‐fixation in 4% paraformaldehyde and 4% sucrose in PBS (pH 7.4) for 24 h, followed by an additional 24‐h equilibration at 4°C in PBS containing 30% sucrose. Brains were embedded in Tissue‐Tek O.C.T. (Sakura, USA) or paraffin. Immunohistochemistry was performed on coronal 40‐µm cryopreserved or 5‐µm paraffin‐embedded sections. The tissue sections were incubated in antigen retrieval buffer (10 mM Tri‐sodium citrate buffer with 0.05% Tween at pH 6.0) at 95°C for 30 min, permeabilized with 0.25% Triton X‐100 and 0.25% Tween 20, and blocked with 3% bovine serum albumin, 10% horse serum, and 0.25% Triton X‐100 at room temperature for 1 h. The sections were then incubated with primary antibody glial fibrillary acidic protein (GFAP) (σ‐Aldrich, Catalog No.: G3893, 1:1,000) 24 to 48 h at 4°C, followed by incubation with the secondary anti‐mouse Alexa 647 (A‐31571, 1:250) for 2 h at room temperature.^23^, ^30^ Sections were then washed and mounted using Vectashield with DAPI (Vector Laboratories, Burlingame, CA, USA). Images were acquired with an Olympus VS200 whole slide scanner system using a 20× UPlanXApo objective (0.8 NA), converted to 16‐bit using Olympus Olyvia software, and analyzed using MetaMorph and ImageJ/Fiji National Institutes of Health software. The analysis was averaged from two to four sections per mouse on background‐subtracted and thresholded images to remove the non‐specific staining. To determine the level of GFAP‐positive gliosis, the percentage of stained area was quantified as the threshold‐stained area divided by the total assigned area in the hippocampus or cortex within the same sections, as we described for GFAP+ and other markers, including Aβ+ staining.^23^, ^30^ For the neurovascular unit (NVU) analysis, the images containing the hippocampal dentate gyrus (DG) region were loaded and converted to 8‐bit format. Brightness and contrast were adjusted to optimize visualization, and similar background subtraction was performed to enhance field clarity. Regions of interest (ROI) encompassing individual NVU at the DG‐CA1 junction were manually delineated using freehand selection tools. A 100 µm^2^ box was drawn surrounding the large vessels (>20 µm diameter, between three and eight vessels per section). Image thresholds were optimized, and the “Analyze Particles” plug‐in function was applied to quantify astrocyte‐positive staining surrounding each NVU.

### 2.6 Immunoblot analysis

Brain tissues were lysed in ice‐cold RIPA buffer composed of 150 mM NaCl, 50 mM Tris‐HCl (pH 7.4), 1% NP‐40, 0.5% sodium deoxycholate, 5 mM EDTA, and 0.1% SDS, supplemented with 2 mM PMSF (Sigma‐Aldrich), protease inhibitor cocktail (1:200, Sigma‐Aldrich), and phosphatase inhibitor (PhosSTOP, Roche). Tissue homogenates were briefly sonicated on ice. Equal amounts of protein were resolved on 4% to 12% Bolt™ Bis‐Tris Plus gels (Invitrogen) and transferred to pre‐cut PVDF membranes (Invitrogen/Thermo Fisher Scientific). APP full‐length (APP‐FL), C‐terminal fragments (APP‐CTF, including α‐CTF and β‐CTF isoforms, and mAICD) were detected using a rabbit polyclonal APP‐CTM1 antibody^31^ (generously provided by Dr. Gopal Thinakaran). mAICD‐CT11 tagged was detected using the rabbit APLP1‐CT11 antibody^31^ (generously provided by Dr. Gopal Thinakaran). GAPDH protein detection was used as a loading control (σ‐Aldrich, Catalog No.: G9795). Fluorescent immunoblots were imaged using the Odyssey Infrared Imaging System (LI‐COR Biosciences) and line‐scan representation using the MetaMorph analysis plug‐in (Molecular Devices). A fixed‐size region of interest was selected around each band, and quantification was performed within the same gel to ensure consistency across samples.

### 2.7 Statistical analysis

Statistical analysis was performed using Prism (GraphPad Software, San Diego, CA, USA, www.graphpad.com). The changes over time are presented as mean ± standard error of the mean (SEM). Two‐way ANOVA with Sidak *post hoc* tests was used to compare changes over time within two groups. The column graphs are presented as a mean ± SD. A one‐way ANOVA Kruskal‐Wallis test was utilized in non‐Gaussian distribution, followed by Dunn's multiple comparisons. An ordinary one‐way ANOVA test was used in Gaussian distribution, followed by Tukey's multiple comparisons. Simple linear regressions were applied to determine Pearson's correlation values. A two‐tailed *p* < 0.05 was considered statistically significant. We provided exact *p* values for both significant and non‐significant conditions when relevant. The significance levels are indicated by asterisks: *p < 0.05, **p < 0.01, ***p < 0.001, and ****p < 0.0001, as compared to control groups.

## 3 RESULTS

Sleep disorders and cognitive impairments are common symptoms of AD. The relationships between these two symptoms in AD patients are not well known. We previously demonstrated that sleep fragmentation could induce cognitive decline, which is strongly negatively correlated with the disruption of BBB permeability.^27^ To explore the interactions between these three parameters, we designed an APP‐CTF construct targeted to the lipid raft, making it competent for constitutive signaling but remaining unperturbed by γ‐secretase, referred to here as the membrane‐tethered APP intracellular domain (mAICD, Figure 1A).^24^ We reported that the expression of mAICD initiates a signaling pathway that involves a direct association between the heterotrimeric Gα_S_ protein.^24^ To assess the functional role of this interaction, we generated a complementary mutant construct (mAICDmutAAA or mutAAA) lacking Gα_S_ binding capability, as well as a the mCtl with the same number of amino acids with a random sequence. In this study, we delivered recombinant AAV in the brain of neonatal 5XFAD mice and non‐transgenic (NTg) littermates to investigate whether mAICD treatment could prevent the memory deficits, cognitive dysfunction, and sleep disturbances that occur in a well‐established murine model of AD.^32^, ^33^ We assessed sleep and cognitive behaviors 6 months after the occurrence of mAICD and mutAAA expression and compared the outcomes to mCtl‐expressing mice. Western blot analysis confirms mAICD expression in the frontal cortex and hippocampus of 40‐week‐old 5XFAD mice using the CT11 Ab, revealing similar levels of expression of endogenous APLP‐1 and mAICD CT11‐tagged proteins (Figure 1B1). These results are consistent with the predicted expression of the AAV construct^34^, ^35^ and with the broad brain detection of the EGFP reporter, as we previously reported.^23^ A difference in APP processing was also observed using the CTM1 Ab, which identifies APP‐CTF metabolites, including mAICD (Figure 1B2). As we would expect from our in vitro cell culture studies,^23^ α‐CTF and β‐CTF accumulations were affected by mAICD expression (Figure 1B2,1B3).

### 3.1 APP‐mediated signaling affects sleep patterns

Sleep was recorded using a non‐invasive, high‐throughput, automated piezoelectric system (Figure 1A). No differences in sleep duration or patterns were observed during the light phase (Figure 1C1). However, 5XFAD mice slept less than NTg littermates (Figure 1D1, *p* < 0.0001), especially during the active period in the dark phase (21.9 ± 0.7% and 31.2 ± 1.9%, respectively). We also observed that NTg littermates expressing the mutAAA variant slept less in the dark phase (26.0 ± 1.5%, *p* = 0.0002), an effect not seen when native mAICD protein was expressed (Figure 1C2,D2). On the other hand, we noted that mAICD expression in 5XFAD mice significantly improved their sleep behavior (*p* < 0.0001; Figure 1C2,D3). The 5XFAD‐mAICD mice had a sleep behavior remarkably similar to that of NTg mice. This effect was not seen in 5XFAD mice expressing the mAICD construct lacking the Gα_S_ interacting site (mutAAA; Figure 1C2), thereby suggesting that the impact of mAICD required the Gα_S_ coupling with the adenylate cyclase and downstream signaling.

### 3.2 APP‐CTF expression correlates with improvement of sleep and cognition

**FIGURE 2:**
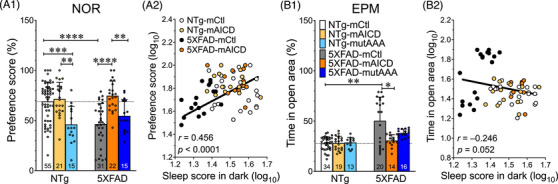
Amyloid precursor protein C‐terminal fragment (APP‐CTF) expression correlates with improvement of sleep and cognition. (A1) Explicit memory was evaluated in 5XFAD and NTg littermates using the Novel Object Recognition (NOR) preference test. Cohorts expressing membrane‐tethered control (mCtl), membrane‐tethered APP intracellular domain (mAICD), and mutated mAICD (mAICD‐mutAAA) variant were compared. (B1) Elevated‐plus maze (EPM) test analysis presented as a percentage of time spent in open arms. Data are shown as mean ± standard deviation (SD). Statistical analysis was performed using ANOVA Kruskal‐Wallis test, followed by Dunn's multiple comparisons. *p < 0.05, **p < 0.01, ***p < 0.001, ****p < 0.0001. (A2, B2) Percentages of sleep in dark, NOR preference scores, and time spent in open arm were converted to log_10_ for NTg and 5xFAD mice expressing mCtl or mAICD. (A2) Correlation between sleep in dark and preference scores is shown (*n* = 73*, r* = 0.456, *Y* = 0.846 × *X + *0.503, *p < *0.0001). (B2) Correlation between sleep and time spent in open arms is shown *(n* = 63, *r* = −0.246, *Y* = −0.377 × X + 2.066, *p* = 0.052).

Poor quality and quantity of sleep appreciably affect memory recollection and consolidation.^36^, ^37^, ^38^ Accordingly, we next explored whether the changes in sleep behavior observed in 5XFAD and NTg mice might impact cognitive function. We used the NOR task to examine whether the mice would exhibit changes in the propensity of recognizing a novel object introduced 1 h after the beginning of the test (Figure 2A). We confirmed that 5XFAD mice expressing the mCtl control viral vector exhibited a lack of interest for novelty in the NOR test as compared to NTg‐mCtl (46.4 ± 3.7% vs 68.9 ± 2.6% *p* < 0.0001; Figure 2A1). We also observed that the brain expression of mAICD in 5XFAD mice rescued the cognitive loss (74.6 ± 3.1%, *p* < 0.0001). This effect was not detected in mice expressing the mutAAA variant (54.6 ± 4.2%), as we previously reported using other cognitive assessment conditions.^23^ As a point of interest, the NTg‐mutAAA mice exhibited a lower preference score (46.0 ± 4.7%) than their NTg‐mCtl and NTg‐mAICD counterpart littermates (mCtl: 68.9 ± 2.6%, *p* = 0.0007, mAICD: 71.3 ± 3.7%, *p* = 0.0014). To investigate the potential contribution of sleep to cognitive performance, we plotted the NOR preference score against the sleep metrics of each mouse (Figure 2A2 and Table S1). We found a significant correlation between both parameters (*r* = 0.456, *p* < 0.0001). The plotted chart displays two distinct populations, indicating a reduction of sleep in the dark and NOR performance in 5XFAD mice. In contrast, better sleep was associated with increased NOR performance scores in NTg‐mCtl and 5XFAD expressing mAICD. We also performed the EPM task to analyze the anxiolytic/impulsivity behavior^39^ (Figure 2B). 5XFAD control mice showed a significant increase in time spent in open arms compared to NTg‐mCtl (50.3 ± 5.3% vs 27.7 ± 1.1%, *p* = 0.004). This effect was not observed in mAICD and mAICDmutAAA variants (Figure 2B1). We found no significant correlations between sleep and EPM's time spent in the open areas (Figure 2B2; see Table S1 for additional analyses). Altogether, our results demonstrate the substantial contribution of APP‐CTF in restoring sleep and memory loss in an AD mouse model. Thus, these data suggest a cAMP/PKA‐dependent contribution of APP‐CTF to the cognitive process.

### 3.3 mAICD expression restores BBB integrity in 5XFAD mice

**FIGURE 3:**
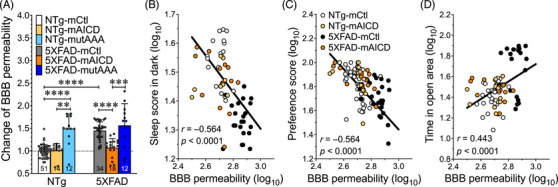
Membrane‐tethered APP intracellular domain (mAICD) expression restores the blood–brain barrier (BBB) integrity in 5XFAD mice. (A) BBB permeability was measured by systemic administration of 4 kDa‐dextran‐FITC in 5XFAD and NTg littermates. Cohorts expressing membrane‐tethered control (mCtl), mAICD, and mutated mAICD (mAICD‐mutAAA) were compared. Data are shown as mean ± standard deviation (SD). ANOVA Kruskal‐Wallis test was performed, followed by Dunn's multiple comparisons. **p < 0.01, ***p < 0.001, ****p < 0.0001. (B) The correlation between BBB permeability and sleep score in the dark is shown (*n* = 64, *r* = −0.564, *Y* = −0.635 × X + 3.208, *p* < 0.0001). (C) Correlation between BBB permeability and Novel Object Recognition preference score is shown (*n* = 114, *r* = −0.564, *Y* = −1.256 × X + 5.212, *p* < 0.0001). (D) Correlation between BBB permeability and time spent in open area in EPM test (*n* = 78, *r* = 0.443, *Y* = 0.848 × X − 0.821, *p* < 0.0001).

To elucidate the mechanisms underlying the observed changes in sleep and cognitive function, we examined the contribution of BBB integrity. We previously reported that the expression of mAICD reduced Aβ burden in the hippocampus of 5XFAD mice.^23^ Sleep disturbances have been shown to increase Aβ production and hamper Aβ clearance through the BBB.^7^, ^10^, ^40^, ^41^, ^42^ The BBB is important in maintaining proper exchange between the brain and the periphery, especially during reparative sleep.^43^, ^44^ To assess BBB permeability, we injected the conjugated small dextran‐FITC protein into the tail vein. Brains were harvested to quantify the change in fluorescence (Figure 3). Higher dextran accumulation was detected in the brains of 5XFAD mice compared to NTg control littermates (1.45‐fold, *p* < 0.0001, Figure 3A). 5XFAD mice expressing mAICD had a lower dextran accumulation in their brains compared to 5XFAD‐mCtl (*p* < 0.0001) and levels similar to those measured in NTg‐mCtl (5XFAD‐mAICD: 1.06‐fold). Expression of mutAAA did not reduce the dextran levels in 5XFAD mice (1.56‐fold). Still, it produced a significant augmentation in NTg mice (1.49‐fold, *p* < 0.0001), thereby suggesting that Gα_S_ interaction with mAICD might contribute to preserving BBB integrity.

To evaluate potential associations between BBB, sleep, and cognitive function, BBB permeability values were plotted against corresponding sleep scores, NOR performance, and EPM open‐arm entry. We observed a highly significant inverse correlation between sleep and BBB permeability (*r* = −0.564, *p* < 0.0001; Figure 3B and Table S1). The reduced sleep observed in 5XFAD mice was associated with increased BBB permeability. In contrast, BBB permeability measures in 5XFAD expressing mAICD were found near the NTg‐mCtl control group values. This interrelated effect was also apparent between BBB permeability levels and NOR preference scores (*r* = −0.564, *p* < 0.0001; Figure 3C), as well as in the EPM open‐arm entry (*r* = 0.443, *p* < 0.0001; Figure 3D). Accordingly, our results confirmed an intimate association between BBB and sleep that might impose consequences on cognitive behaviors, whereby APP‐CTF signaling could play a distinctive role in influencing AD pathology.

### 3.4 Sex effect associated with BBB permeability, sleep, and cognitive function

**FIGURE 4:**
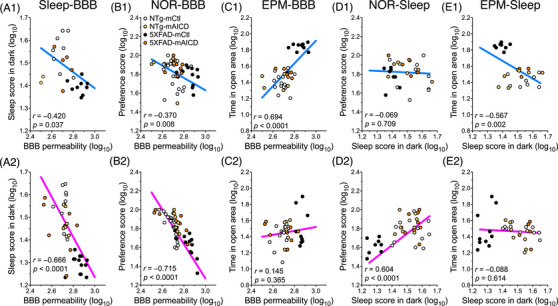
Sex effect associated with blood–brain barrier (BBB) permeability, sleep, and cognitive function. Correlation plots are shown for NTg and 5XFAD mice expressing membrane‐tethered control (mCtl) or membrane‐tethered APP intracellular domain (mAICD). The log values of BBB permeability were plotted for each animal against (A) the sleep score in the dark, (B) against the Novel Object Recognition (NOR) preference score, and (C) against the time spent in the open arms of the Elevated‐plus maze (EPM) test. The log values of the sleep score in the dark were plotted for each animal (D) against the NOR preference score and (E) against the time spent in the open arms of the EPM test. (A1, B1, C1, D1, E1) Correlation plots in males are shown in the upper panels, whereas (A2, B2, C2, D2, E2) the correlation plots in females are shown in the lower panels. (A) Males and females exhibit a negative correlation between the BBB permeability and their sleep scores in dark (males: *n* = 25, *r* = −0.420, *Y* = −0.355 × X + 2.453, *p* = 0.037; and females: *n* = 39, *r* = −0.666, *Y* = −0.885 × X + 3.889, *p* < 0.0001). (B) The correlation between NOR preference scores and the BBB was more significant in females (females: *n* = 54, *r* = −0.715, *Y* = −1.787x + 6.625, *p* < 0.0001; and males: *n* = 46, *r* = −0.370, *y* = −0.680x + 3.668, *p* = 0.008), (C) while the males showed a better correlation with the EPM scores (males: *n* = 37, *r* = 0.694, *Y* = 1.409 × X − 2.311, *p* < 0.0001; and females: *n* = 41, *r* = 0.145, *Y* = 0.241 × X + 0.796, *p* = 0.365). (D) Correlation is shown between sleep during dark phase and NOR preference score in males (*n* = 32, *r* = −0.069, *Y* = −0.082 × X + 1.942, *p* = 0.709) and females (*n* = 41, *r* = 0.604, *Y* = 1.233 × X − 0.103, *p* < 0.0001). (E) Correlation is shown between sleep during the dark phase and time spent in open arms in the EPM test in males (*n* = 28, *r* = −0.567, *Y* = −1.098 × X + 3.202, *p* = 0.002) and females (*n* = 35, *r* = −0.088, *Y* = −0.105 × X + 1.620, *p* = 0.614).

Next, we performed a detailed analysis to distinguish potential sex‐related differences in each condition (Figures 4, S1, Table S1). Although there were no sex differences among the NTg mice, such differences emerged among 5XFAD mice in terms of performance during the NOR (Figure S1A) and the EPM (Figure S1B) tasks. The effects on NOR performance and sleep duration were more noticeable in females expressing mAICD (Figure S1A, *p* < 0.001; and Figure S1C, *p* < 0.0001). Although the change in the BBB was comparable in both sexes (Figure S1D), we observed that sleep measures correlated more significantly with BBB permeability in female mice (*r* = −0.420, *p* = 0.037, *n* = 25; and *r* = −0.666, *p* < 0.0001, *n* = 39; males and females, respectively; Figures 4A1 and 4A2). We also plotted individual BBB permeability measures against NOR preference scores and found a robust correlation between the performance scores and BBB permeability in female mice (*r* = −0.715, *p* < 0.0001, *n* = 63; Figure 4B2). This effect was not as pronounced in the male counterparts (*r* = −0.370, *p* = 0.008, *n* = 51; Figure 4B1). Moreover, we plotted BBB permeability against the percentage of time spent in the open arm of the EPM. Comparisons between mCtl and mAICD cohorts revealed a robust correlation in male mice (*r* = 0.694, *p* < 0.0001, *n* = 37; Figure 4C1), which was not apparent in female mice (*r* = 0.145, *p* = 0.365, *n* = 41; Figure 4C2). Analysis between sleep and NOR scores indicated that female mouse performances were linked to a better sleep score (*r* = 0.604, *p* < 0.0001, *n* = 41, Figure 4D2), an effect that was not observed in male mice (*r* = −0.069, *p* = 0.709, *n* = 32; Figure 4D1). On the other hand, we found that the time spent in the open arm was more interconnected with reduced sleep in male cohorts (*r* = −0.567, *p* = 0.002, *n* = 28; Figure 4E1) but not in females (*r* = −0.088, *p* = 0.614, *n* = 35; Figure 4E2). The scatter plots indicate that 5XFAD mice expressing mAICD exhibited associative behaviors similar to those observed in NTg‐mCtl cohorts. Altogether, our results indicate that mAICD expression has greater consequences for restoring sleep in female mice, which parallel improvements in BBB permeability and cognitive performance.

### 3.5 APP‐mediated signaling influences astrogliosis

**FIGURE 5:**
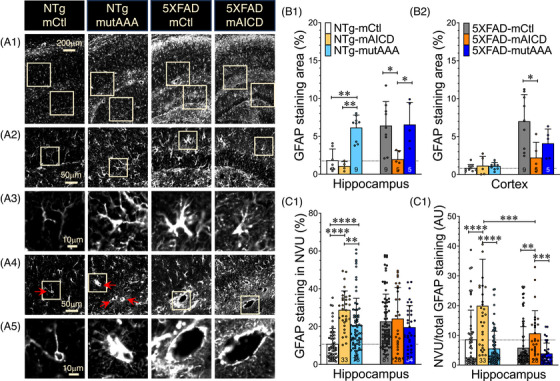
Amyloid precursor protein (APP)‐mediated signaling influences astrogliosis. Astrogliosis was examined in brain sections of NTg and 5XFAD mice expressing membrane‐tethered control (mCtl), membrane‐tethered APP intracellular domain (mAICD), and mAICD‐mutAAA variant, using the glial fibrillary acidic protein (GFAP) monoclonal antibody. Scanned images were acquired through a 20× objective using the Olympus VS200 scanner system. (A1) Representative images of GFAP immunostaining in the hippocampus are shown for NTg‐mCtl, NTg‐mAICDmutAAA, 5xFAD‐mCtl, and 5xFAD‐mAICD adeno‐associated virus (AAV)‐expressing mice. Enlarged images of dentate gyrus (DG) and CA1 areas are displayed (A2 and A4, respectively). (A3) Gliosis stages are shown through a change in the astrocyte morphology and accumulation of GFAP‐positive staining surrounding vasculature (A4 red arrows, and A5 enlarged images). (B) The percentages of GFAP‐positive immunolabeling areas were quantified with Fiji ImageJ and MetaMorph software in the (B1) hippocampus and the (B2) cortex of NTg and 5XFAD brain slices. (C1) GFAP‐positive gliosis was quantified in the neurovascular unit (NVU) located at the junction of the molecular layer of the DG and the lacunosum moleculare stratum of the hippocampus. (C2) Results are reported as a ratio between GFAP staining surrounding NVU divided by the total staining in the hippocampus. Data are shown as mean ± standard deviation (SD). Statistical analysis was performed using a one‐way ANOVA Kruskal‐Wallis test, followed by Dunn's multiple comparisons (B), or an ordinary one‐way ANOVA test, followed by Tukey's multiple comparisons (C). *p < 0.05, **p < 0.01, ***p < 0.001, ****p < 0.0001.

The important relationship between sleep and BBB permeability led us to explore the possibility that certain components of the NVU might be affected. Alterations of BBB integrity can contribute to various pathological mechanisms that involve the astrocytic response (reviewed elsewhere^2^, ^15^, ^45^). Astrocytes are recognized as playing an important role in regulating the BBB exchange of neurotoxins, as well as allowing for Aβ clearance. Astrocyte end‐foot processes express GFAP, a marker that is highly enhanced in the brain of AD patients as well as in several AD mouse models.^46^ The astrocyte end‐foot processes are dynamic structures that enwrap the brain microvasculature, and their disruption can lead to BBB damage.^2^, ^15^, ^45^ We followed up on the idea that astrocytic response might be altered distinctively in our mouse cohorts exhibiting BBB changes. We used the GFAP astrocyte marker to determine whether mAICD expression in the brain might affect the level of expression of activated astrocytes (Figure 5). Although GFAP staining was intensified in 5XFAD mice compared to the NTg mice, we also observed distinct differences in astrocyte morphology and distribution between groups (Figure 5A1,A2). The activated astrocytes in 5XFAD‐mCtl brain sections displayed a typical enlargement of the main branches. In contrast, the astrocytes in 5XFAD mice expressing mAICD showed diminished protruded branches (Figure 5A3). On the other hand, NTg‐mCtl mice exhibited a more quiescent morphology, while NTg mice expressing the mAICDmutAAA variant showed more astrocytic ramifications. We noticed more staining surrounding the small blood vessels, as indicated by the arrows (Figure 5A4,A5). More staining was also detected surrounding the large NVU in 5XFAD mice.

The percentage of GFAP‐positive area was quantified in the hippocampus (Figure 5B1) and the cortex (Figure 5B2) of mice expressing mCtl, mAICD, or mAICDmutAAA. The quantitative analysis revealed that NTg mice expressing mutAAA exhibited significantly higher overall GFAP immunoreactivity in the hippocampus compared to NTg‐mCtl (*p* = 0.007) and NTg‐mAICD (*p* = 0.009). This effect was not seen in the cortical area (Figure 5B2). We also noted in 5XFAD mice expressing mAICD a significant decrease of GFAP expression in the cortex (*p* = 0.040) and in the hippocampus (*p* = 0.027), which was not detected in the counterpart mutAAA group (Figure 5B1). We quantified the immunostaining in large NVU located at the junction of the molecular layer of the DG and the lacunosum–moleculare stratum of the hippocampus (Figure 5C). GFAP immunostaining in NVU was increased in 5XFAD mice regardless of the AAV injection groups (Figure 5C1). Interestingly, we observed an increase in GFAP+ staining in NTg mice expressing either mAICD or mAICDmutAAA, though to a lesser extent with mutAAA (2.67‐fold vs 1.92‐fold, respectively, *p* = 0.003). When analyzing the NVU staining over the entire hippocampal area, mAICD‐expressing mice exhibited increased NVU‐associated GFAP‐covered ratio area (Figure 5C2), suggesting that mAICD may promote a redistribution of astrocytes around the NVUs. The mAICD effect was significantly lower in 5XFAD mice compared to NTg (19.9 ± 2.7 vs 10.6 ± 1.4 increased ratio, *p* = 0.0004). The ratio levels were similar across the other experimental groups, including the mutAAA variant, which supports the idea that mAICD's effect involves Gα_S_ downstream signaling. Overall, the observed changes in GFAP immunoreactivity may indicate a potential link between APP‐CTF expression and alterations in the BBB integrity observed under these experimental conditions.

## 4 DISCUSSION

Perturbed sleep is considered an early indicator of memory decline and a risk factor for AD.^6^, ^36^, ^47^ It has been reported that the ability to maintain homeostatic Aβ production and clearance in AD is hampered by chronic sleep disturbances, leading also to the exacerbation of cerebral Aβ burden.^47^, ^48^ Aβ levels in the brain are closely linked to the circadian cycle,^7^, ^10^, ^49^ and its accumulation leads to sleep disturbance.^10^, ^47^, ^48^ The mechanisms underlying these manifestations are poorly understood (reviewed elsewhere^11^). Our findings indicate APP‐mediated signaling tampers memory deterioration associated with sleep disturbances in an AD mouse model. We determined that the downstream coupling of APP‐CTF to adenylate cyclase could prevent memory deficits. Our study also highlights the interrelationships between the amount of sleep, the degree of BBB leakage, and cognitive performance. It has been reported that prolonged wakefulness impairs the cAMP‐dependent pathway, which is detrimental to memory consolidation.^50^, ^51^ In this context, we report the protective effects of mAICD expression in an AD mouse model, leading to heightened sleep magnitude, memory improvement, and BBB permeability recovery. We also pinpoint the critical role of cAMP signaling throughout the adenylate cyclase coupling within the APP‐CTF sequence, as we demonstrated that the mAICDmutAAA variant lacking the Gα_S_ interaction site did not rescue the described defects. Therefore, current studies are shedding light on the importance of APP‐CTF as a putative molecular target underlying the degenerative changes recorded herein.

Our findings are consistent with our previous work demonstrating that enhanced APP‐CTF in an AD mouse model, through mAICD overexpression since birth, can reduce Aβ burden in the hippocampus and rescue explicit memory impairments.^23^ It is well documented that Gα_S_ coupling hampers Aβ production,^52^, ^53^, ^54^ which may thereby preserve sleep architecture and function. Observations from circadian and synaptic biology studies demonstrate that Aβ is released upon increased brain activity, including during wakefulness, as was mutually noticed in AD mouse models and patients.^4^, ^5^, ^10^, ^40^, ^41^, ^55^, ^56^ Whether reduced sleep and Aβ accumulation in AD initiate BBB leakage remains an open question. Recent work from the Holtzman group indicates that ApoE might contribute to this process^57^ (see also Sadleir and Vassar^58^). Our results raise the possibility that APP‐CTF might also participate in maintaining BBB integrity and facilitating Aβ clearance during sleep. Many studies have stressed that the prototypical cerebrovascular lesions in neurodegenerative diseases, also seen in AD, appear to frequently coexist with BBB breakdown and dysfunction.^2^, ^18^, ^45^, ^59^ The NVU becomes progressively dysfunctional in AD, and each of its components may undergo changes that contribute to neuronal injury and cognitive deficits. Nonetheless, data from barrier leakage studies in 5XFAD mice lead to quite complex and somewhat divergent conclusions.^60^, ^61^, ^62^ In our study, we used the conjugated dextran‐FITC 3‐ to 5‐kDa molecule, which reflects only the apparent permeability through the tight and adherent junctions rather than being linked to other pathological courses.^29^ BBB dysfunction in AD influences Aβ clearance and endothelial transport, impairs endothelial cell and pericyte functions, affects tight junction integrity, activates glial cells, and facilitates the recruitment of leukocytes in the brain and activation of microglia (reviewed in Zenaro et al.^45^). Our findings suggest that mAICD expression might contribute to that process by preventing extensive BBB leakage, which was also associated with a reduction in astrogliosis that appeared to involve interaction with the cAMP/PKA pathway. The literature supports a fundamental role of adenylate cyclase activation in regulating BBB integrity through nearby communication with astrocytes and microglia, two cellular units engaged during sleep and wakefulness states (reviewed elsewhere^63^, ^64^, ^65^). The close interaction of astrocytes with the BBB also deserves attention,^2^, ^15^ particularly since glymphatic clearance is more active during sleep but impaired in AD.^7^, ^41^ The mechanisms linking sleep disruption/sleep loss to BBB breakdown in AD remain to be determined.

Among the salient findings of this study, the differences between male and female mice are particularly worthy of mention. Women are two to three times more likely to develop AD after the age of 65 years.^1^, ^66^, ^67^, ^68^ Sex differences in several behavioral tests have been reported in patients with AD, whereby men suffering from AD seem to exhibit more apathy, agitation, and behavioral disruption. In contrast, women are more likely to present depressive symptoms, reclusiveness, emotional lability, delusions, and affective and manic symptoms.^67^, ^68^, ^69^ The frequently observed differences in the prevalence of AD between sexes were assumed to reflect the longer lifespan in women. However, several proposed origins have been described in human and mouse models (reviewed elsewhere^70^). As a point of interest, a sex effect was also observed in cognitive performance related to sleep quality.^71^ Although a small cohort of individuals was used, women were more impaired in their spatial orientation performances with limited sleep. In contrast, poor sleep did not significantly affect men's scoring performances. Similarly, we found evidence in our study showing different sleep patterns among 5XFAD mice. The female mice slept less than their male counterparts during the dark phase, which was also reported by others.^72^, ^73^ We also observed a stronger association between spatial cognitive performance and sleep duration in female mice, with the female mice being affected more by memory impairment in reduced sleep conditions, which was not noticed in males. The close relationship between cognitive performance and leakage of the BBB, especially in female mice, also emphasizes the sex‐dependent impact of the vascular unit disruption in AD.

Finally, and perhaps most importantly, our results highlight that neonatal expression of mAICD afforded substantial protection of the anticipated functional changes in our AD mouse model. In the context of this recognized mouse model of neurodegenerative disease, mAICD expression rescued deficits in the vascular unit, cognition, and sleep behavior, suggesting that APP‐CTF may have translational relevance beyond what has been previously acknowledged.^59^, ^74^ The requirement for mAICD interaction with Gα_S_, which entails subsequent activation of the adenylate cyclase and cAMP‐dependent downstream pathways, was abolished by the mutAAA variant, highlighting the mechanistic aspects of mAICD function. Altogether, our results demonstrated the critical role of APP‐mediated signaling in regulating sleep‐associated brain circuitry and memory proficiency, which may have consequences for AD etiology.

Now that we have identified a role for APP‐mediated signaling in restoring BBB integrity and sleep, we will need to determine whether a potential translational intervention can be successfully implemented by targeting other AD models or a later stage of AD pathology. The influence of adenylate cyclase on BBB integrity has been documented but not fully understood.^63^ A better characterization of BBB leakage emerged as a significant technical limitation, as this study employed only the 3‐ to 5‐kDa dextran probe. Use of various markers will be essential to delineate alterations in the NVU. Tight junction proteins, extracellular matrix proteins, endothelial cells, pericytes, and transcytosis markers are just a few of the NVU components that can affect BBB integrity.^74^, ^75^ It will be essential to establish whether the observed effect is due to the expression of mAICD in specific cell types. The CAG promoter and AAV 2/8 capsid employed in our study target multiple cell types. Although we observed that activated astrocytes were detected closer to the NVU when mAICD is expressed, the cellular mechanisms underlying this observation are unknown. We cannot rule out contributions from neurons, microglia, pericytes, or endothelium to the observed outcomes. This conundrum can be addressed using cell‐type‐specific promoters in AAV constructs to direct mAICD expression to specific cell types. The sex differences observed across our studies also pose limitations, as genetic background may influence our results; however, patient studies support our findings. We also acknowledge that the estrous cycle and hormonal changes might contribute to the disparity in our results, underscoring multiple factors that can affect BBB homeostasis.^74^ Conducting deeper signaling studies using proteomics, cAMP/PKA inhibitors, or downstream effector analyses will provide mechanistic elucidation of the significance of other pathways involved.

## CONFLICT OF INTEREST STATEMENT

No conflict of interest to declare. Author disclosures are available in the Supporting Information

## CONSENT STATEMENT

Not applicable for this study. Consent was not necessary.

## Supporting information



Supporting Information

Supporting Information

Supporting Information

Supporting Information

Supporting Information
