# Supplementary material for: APP‐mediated intracellular signaling rescues sleep impairment and blood–brain barrier leakage in Alzheimer's disease mouse model
Source: Alzheimers Dement. 2026 Feb 3;22(2):e71134. doi: 10.1002/alz.71134 (PMC12865326; doi:10.1002/alz.71134)
Supplement: Supplementary file 3 — Supporting Information [file ALZ-22-e71134-s005.docx]

**Table S1: Pearson's correlation analysis**
