# Supplementary material for: APP‐mediated intracellular signaling rescues sleep impairment and blood–brain barrier leakage in Alzheimer's disease mouse model
Source: Alzheimers Dement. 2026 Feb 3;22(2):e71134. doi: 10.1002/alz.71134 (PMC12865326; doi:10.1002/alz.71134)
Supplement: Supplementary file 5 — Supporting Information [file ALZ-22-e71134-s004.docx]

**Figure S1: Sex differences affect sleep, BBB, and cognitive behaviors.** Preference score during the NOR test, n=6-28 **(A)**, time spent in open arms during the EPM test, n=6-18 (**B**), percent sleep during the dark phase, n=8-13 (**C**), and change in the BBB permeability, n=6-27 (**D**) are reported in males (blue) and females (pink). Data are shown as mean ± SD. Statistical analysis was performed using a one-way ANOVA Kruskal-Wallis test, followed by Dunn's multiple comparisons. * *p* < 0.05, ** *p* < 0.01, *** *p* < 0.001, **** *p* < 0.0001.
